# Neoadjuvant chemotherapy with biosimilar trastuzumab in human epidermal growth factor receptor 2 overexpressed non-metastatic breast cancer: patterns of use and clinical outcomes in India

**DOI:** 10.3332/ecancer.2021.1207

**Published:** 2021-03-19

**Authors:** Anjana Joel, Josh Thomas Georgy, Divya Bala Thumaty, Ajoy Oommen John, Raju Titus Chacko, Grace Rebekah, Elanthenral Sigamani, Jagan Chandramohan, Marie Therese Manipadam, Anish Jacob Cherian, Deepak Thomas Abraham, Paul Mazhuvanchary Jacob, Patricia Sebastian, Selvamani Backianathan, Ashish Singh

**Affiliations:** 1 Department of Medical Oncology, Christian Medical College, Vellore 632004, India; 2 Department of Biostatistics, Christian Medical College, Vellore 632004, India; 3 Department of Pathology, Christian Medical College, Vellore 632004, India; 4 Department of Endocrine Surgery, Christian Medical College, Vellore 632004, India; 5 Department of Radiation Therapy, Christian Medical College, Vellore 632004, India

**Keywords:** biosimilar, breast cancer, non-metastatic, neo-adjuvant, short duration, trastuzumab

## Abstract

**Background:**

Human epidermal growth factor receptor 2 (HER2)-positive breast cancer is associated with poor prognosis and access to anti-HER2 treatment is still a challenge in lower-middle income countries. The availability of the biosimilar trastuzumab has improved access by lowering the costs. We report the pattern of use of neoadjuvant ± adjuvant trastuzumab and outcomes in patients with HER2-positive non-metastatic breast cancer treated with regimens incorporating shorter durations of therapy and the use of the biosimilar trastuzumab compared to the innovator.

**Methods:**

We conducted a retrospective analysis of patients with non-metastatic HER2-positive breast cancer treated with neoadjuvant ± adjuvant trastuzumab (innovator (*n* = 34 (33%)) and biosimilar (*n* = 70 (67%)) manufactured by Biocon Biologics) with chemotherapy. Information regarding chemotherapy regimens, duration of trastuzumab use (≤12 weeks and >12 weeks), pathological response (Miller Payne grade), disease free survival (DFS), overall survival (OS) and safety data were collected from electronic medical records.

**Results:**

A total of 135 patients were analysed with a median age of 51 years (range: 23–82); of these, 57% were postmenopausal, 31.8% were hormone receptor positive and 62.9% had stage III disease. The overall pathological complete response (p-CR) in both breast and axilla increased to 37.6% in patients treated with trastuzumab preoperatively as compared to 22.2% in patients who did not receive any trastuzumab. Patients receiving innovator trastuzumab and biosimilar trastuzumab showed a p-CR of 28.5% and 41.7%, respectively. At a median follow-up of 42 months (range: 3–114), there were 18 relapses and 11 deaths. The 3-year DFS was 87.1% and OS was 92.2%. Cardiac dysfunction developed in 4 of 78 (5.1%) evaluable patients.

**Conclusion:**

Access to anti-HER2 therapy in the treatment of non-metastatic HER2-positive breast cancer in resource-constrained settings has improved significantly with the availability of the biosimilar trastuzumab. Imbalances in patient profiles at baseline in routine clinical practice led to inconclusive outcomes of ≤12 weeks versus >12 weeks trastuzumab treatment. However, on the basis of historical data, patients could be offered shorter duration of trastuzumab when a standard 1-year treatment of adjuvant trastuzumab is not feasible in resource-constrained settings. The p-CR using the biosimilar trastuzumab in neoadjuvant treatment has been observed to be comparable to the innovator trastuzumab.

## Background

The non-remunerable expenditure for cancer therapies in the developing world often highlights the glaring imbalance in the differential access to these therapies. Human epidermal growth factor receptor 2 (HER2)-positive breast cancer accounts for 26%–50% of all breast cancers in the Indian population and is associated with an inferior survival outcome mostly attributable to lack of access to an appropriate anti-HER2 therapy [1]. The use of trastuzumab has resulted in an improvement in disease free survival (DFS) and overall survival (OS) of patients with HER2-positive non-metastatic breast cancer which has been observed to be consistent even on a long-term follow-up [2–5]. The current standard of care is 1 year of adjuvant trastuzumab treatment [6].

Shorter durations of trastuzumab have also been evaluated in several studies [7–13]. In the FINHER study, 9 weeks of adjuvant trastuzumab improved distant DFS when compared with no trastuzumab [8]. Few randomised studies of shorter duration trastuzumab; 6 months (PHARE) and 9 weeks (Hellenic Oncology Research Group, Short-HER and SOLD) failed to prove their non-inferiority when compared to 12 months in terms of DFS [10–13]. However, the absolute differences in DFS between shorter duration and 1-year trastuzumab arms were small (1%–2%) across these studies [10, 13]. A subgroup analysis of the PHARE study suggested that the additional benefit of 1 year of trastuzumab over 6 months may be irrelevant in very low-risk subgroup [14]. Subsequently, the PERSEPHONE study demonstrated non-inferiority of 6 months versus 1-year adjuvant trastuzumab treatment [7]. Pathological complete response (p-CR) is an important surrogate marker in HER2-positive breast cancer which leads to reduced recurrence rate and improved rates of DFS and OS [15, 16]. The delivery of trastuzumab in non-metastatic breast cancer in low- and middle-income countries is a challenge because of limited access, resource constraints and high cost due to a non-reimbursable, out-of-pocket expenditure pattern of healthcare provision [17, 18]. A prior analysis from India has shown that only 35.8% of eligible HER2-positive patients could receive trastuzumab as part of routine care, because of financial constraints [19]. Intent of this retrospective analysis was firstly, to identify if in resource constraint setting ≤12 weeks is useful over >12 weeks of trastuzumab treatment. Secondly, if affordable biosimilar could be used in place of innovator to get similar outcomes in routine clinical practice.

Here, we report the pattern of use of neoadjuvant trastuzumab (innovator and biosimilar), p-CR rates and survival outcomes along with a sufficient follow-up period helpful to draw meaningful conclusions.

## Patients and methods

### Design and study population

We analysed the outcomes among all patients who received treatment for HER2 overexpressed breast cancer at our institution from October 2010 to August 2017. Consecutive patients diagnosed with HER2 overexpressed operable breast cancer between the years 2010 and 2017 were included in the analysis. Data was collected from the electronic medical records (EMR) and patient charts. Follow-up data was extracted from the EMR and attempts were made to contact each patient by telephone. This retrospective study was approved by the institutional review board (IRB number: 9297) on 5 February 2015.

Patients with non-metastatic T1–T4, N0–N3 and M0, HER2-positive breast cancer were included in this analysis. HER2-positivity was defined as either immunohistochemical score of 3+ and/or amplification by fluorescent *in situ* hybridisation using CAP 2007 criteria [20].

### Treatment

The chemotherapy consisted of four cycles of anthracycline based chemotherapy: epirubicin (90 mg/m^2^) or doxorubicin (60 mg/m^2^) with cyclophosphamide (600 mg/m^2^) given in 3-weekly or dose dense 2-weekly schedule and either 12 doses of weekly paclitaxel (80 mg/m^2^) or 4 doses of dose dense 2-weekly paclitaxel (175 mg/m^2^) with or without trastuzumab in the neoadjuvant setting in either sequence. Patients with oestrogen receptor and/or progesterone receptor positive tumours received standard adjuvant endocrine therapy (aromatase inhibitor in post-menopausal and tamoxifen in pre-menopausal patients) continued for at least 5 years. Locoregional treatment including surgery and radiotherapy was performed in all patients in this analysis.

### Study assessments and analysis

The objective of our analysis was to evaluate p-CR rates, DFS, OS and toxicity in this cohort of patients. DFS was defined as the interval between histopathological diagnosis and the first DFS event. Patients who did not experience a DFS event at the time of data cut-off or last follow-up were censored. The DFS events during follow-up included: an ipsilateral or contralateral invasive breast cancer, recurrence at any site, a second invasive cancer at any site or death due to any cause, whichever occurred first. The other endpoints were OS (time interval between histopathological diagnosis and death due to any cause), p-CR (ypT0/is, N0; defined as an absence of invasive cancer in the breast and lymph nodes in the surgical specimen) in those undergoing neoadjuvant therapy, and adverse event rate including cardiac toxicity. DFS and OS were estimated by the Kaplan–Meier method using the IBM SPSS® software (version 23.0).

## Results

### Patients and treatment

The medical records of 135 patients who were diagnosed with operable HER2-positive breast cancer between October 2010 and August 2017 at our centre were evaluated with a data cut-off in April 2020. The median age was 51 years (range: 23–82); 57% were postmenopausal, 31.8% were hormone receptor positive and 62.9% had stage III disease.

The baseline characteristics of the patients are shown in Table 1. Out of 135 patients, 104 (77%) received trastuzumab (69 neoadjuvant ± adjuvant and 35 adjuvant). Seventy (67%) patients received biosimilar trastuzumab manufactured by Biocon Biologics, while 34 (33%) received the innovator trastuzumab. Ninety-nine (73%) patients received chemotherapy before surgery. Thirty-one (23%) were treated with chemotherapy alone, as they could not receive trastuzumab due to financial constraints.

The patients were grouped for survival analysis into three groups based on the duration of use of trastuzumab (Table 1). Thirty-one (22.9%) patients received trastuzumab for a duration shorter than 12 weeks, 73 (54.0%) patients were treated for more than 12 weeks and 31 (23%) did not receive any trastuzumab. The groups were not balanced with respect to their baseline disease stage with 74% of patients having stage III disease in the ‘less than 12 weeks’ trastuzumab cohort. The chemotherapy details are provided in Table 2.

### Response, disease-free survival and overall survival

Eighty-seven patients were evaluated for p-CR, of which 69 received trastuzumab (21 – innovator trastuzumab and 48 – biosimilar trastuzumab) and 18 received only chemotherapy. The overall p-CR rate among patients who received neoadjuvant trastuzumab with chemotherapy was 37.6%. Patients receiving innovator and biosimilar trastuzumab showed a p-CR of 28.5% and 41.7%, respectively. Patients who only received the chemotherapy had a p-CR rate of 22.2% (Table 3).

The median follow-up was 42 months (range: 3–114 months). Follow-up data was available for 78.5% patients for up to at least 2 years and for 61.4% patients for up to 3 years. There were 18 DFS events and 12 OS events. As we were unable to follow-up nearly 40% of patients for 3 years, the number of events in DFS and OS data is probably underestimated.

**Figure 1.**
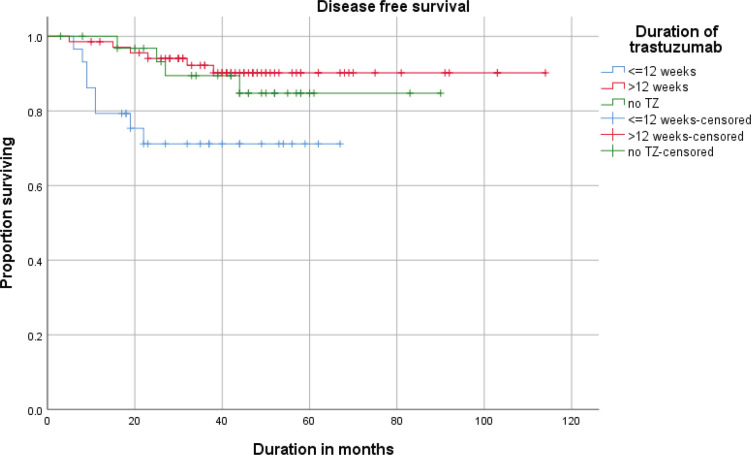
Disease free survival (DFS) of patients receiving neoadjuvant ±adjuvant or adjuvant trastuzumab (≤12 weeks vs >12 weeks).

**Figure 2.**
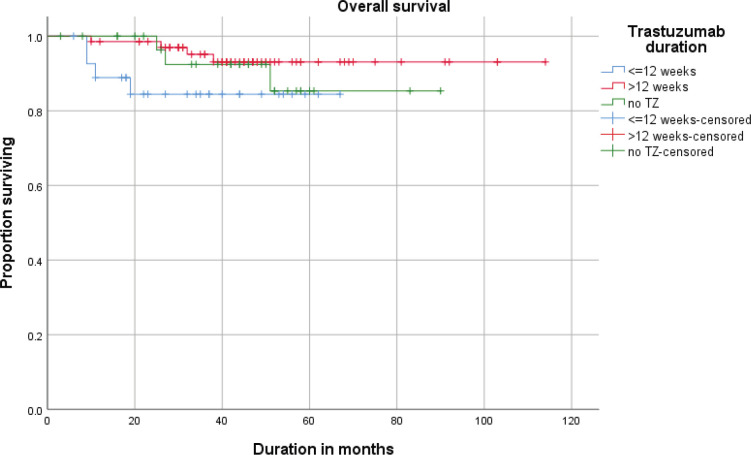
Overall survival (OS) of patients receiving neo adjuvant ±adjuvant or adjuvant trastuzumab (≤12 weeks vs >12 weeks).

The estimated (Kaplan–Meier analysis) 3-year DFS was 87.1% and the 3-year OS was 92.2%. DFS and OS for <12 weeks and >12 weeks are given in Figures 1 and 2.

### Adverse events

The toxicity data was collected from the medical records. Grade III/IV peripheral neuropathy was seen in four patients among whom neuropathy was documented. A cardiac event was defined as a reduction in left ventricular ejection fraction of ≥10% from baseline. At the time of analysis, 4 of 78 (5.1%) evaluable patients had experienced an asymptomatic decline in the ejection fraction. No patient had symptomatic cardiac dysfunction and no patient died due to cardiotoxicity. Long-term follow-up data for cardiac dysfunction was not available.

## Discussion

Biosimilar trastuzumab has been approved for use in advanced breast cancer and has significantly improved access to anti-HER2 therapy in India. This is the first report of its efficacy in a neoadjuvant setting in non-metastatic breast cancer.

Neoadjuvant trastuzumab with chemotherapy improves p-CR, reduces the risk of relapse, disease progression and death. Gianni *et al* [16] in the NOAH study have reported a p-CR rate of 43% versus 22% with trastuzumab in neoadjuvant setting compared to chemotherapy alone, respectively, in patients with newly diagnosed locally advanced breast cancer. In another study, Minckwitz *et al* [21] reported that sequential anthracycline-taxane-based chemotherapy in combination with trastuzumab resulted in a p-CR of 40% compared to a p-CR of 17% with the chemotherapy alone. In this real-world scenario, patients receiving neoadjuvant chemotherapy with any of the two trastuzumab’s showed an overall p-CR rate of 37.6%, with innovator and biosimilar trastuzumab resulting in a p-CR of 28.5% and 41.7%, respectively. Our cohort of patients had higher p-CR rates, irrespective of the number of cycles of neoadjuvant trastuzumab provided, as compared to prior smaller cohorts reported previously from India (34.2% and 36.3%) [22, 23]. While, OS and progression-free survival (PFS) could not be calculated for biosimilar versus innovator because of missing data and low sample size. However, as per recent meta-analysis published in 2020, achieving p-CR following neoadjuvant chemotherapy (NACT) is correlated with improved event free survival and OS particularly in HER2 +ve and triple negative breast cancer [24].

The present report, which includes 64% locally advanced breast cancer patients, shows a 3-year DFS of 87.1% and an OS of 92.2%, using an anthracycline and taxane based chemotherapy in combination with a shorter duration treatment with trastuzumab. This is in contrast with the DFS and OS outcomes after 1 year of trastuzumab treatment as reported in the HERceptin Adjuvant study and the combined NSABP B31 and NCCTG N9831 results at 4 years, ranging from 79%%–85% and 89%%–91%, respectively [3, 25].

Our cohort of women included a higher proportion of patients with advanced breast cancer as compared to the prior reported trials, which had a larger proportion of early breast cancers. Our follow-up duration, however, is shorter at 42 months and only two-thirds of our patients could be followed-up for 3 years. This is due to the fact that the patients preferred to report at a local hospital closer to their homes for a follow-up.

Since majority of these women presented with locally advanced breast cancer, it is imperative to compare our results with the pertinent subgroup analyses from randomised trials using adjuvant trastuzumab. In the latest joint analysis report of the NCCTG N9831 and NSABP B-31 trials, the 4-year DFS of patients with tumour size greater than 5 cm was 52% in the control group and 78% with 1 year of trastuzumab treatment [5]. In a smaller phase II randomised study (E2198) comparing 12 weeks of trastuzumab treatment with 1 year treatment in patients with stage II and IIIA disease, the 5-year DFS was 74% and 78% and OS was 91% and 89%, respectively. This study did not find a significant difference in the two groups, though it was inadequately powered for significance [26]. Recent retrospective study in Ontario reported that less than 1 year of trastuzumab treatment increases risk of relapse and death [27]. The phase III randomised trials with shorter duration of trastuzumab treatment (ranging from 9 weeks to 6 months), including the more recent PERSEPHONE trial, report non-inferiority to 1-year trastuzumab treatment [7, 8, 10, 11, 13]. Although the optimal duration of adjuvant trastuzumab is still debatable, 1 year remains the standard of care as of today. The results of our study suggest that a regimen with a shorter duration of trastuzumab treatment improves outcomes compared to historical controls of patients not receiving trastuzumab. In the real-world setting of low middle-income groups, inclusion of biosimilar trastuzumab, albeit for a shorter duration, in advanced non-metastatic patients can lead to improved p-CR and OS.

In the clinical trials using shorter duration of trastuzumab (6 months and 9 weeks), the incidence of cardiac dysfunction was 3.4% and 5% and that of congestive heart failure around 0.5%; in contrast to 4% in the NSABP31 study where the treatment was of 1 year duration [10, 13, 28]. Retrospective analyses among patient cohorts in routine clinical practice describe the incidence of symptomatic heart failure at 3% and 6.6% among patients receiving sequential therapy [29, 30]. Also, in this real-world cohort, the incidence of a major cardiac event was higher (22.1% versus 17% versus 6.5%) among patients receiving sequential taxanes/trastuzumab followed by anthracycline versus those receiving trastuzumab without anthracyclines versus those receiving anthracyclines without trastuzumab [30]. The incidence of cardiac toxicity in our patient cohort receiving shorter duration of trastuzumab was as expected at 5.1% (4 patients). The overall safety profile was similar in patients who received trastuzumab. The higher incidence of cardiac failure in our analysis could be attributable to higher incidence of comorbid conditions including cardiac predisposition in a real-world population compared to those observed in clinical trials. This may also be attributable to a higher proportion of patients receiving sequential anthracycline and trastuzumab (64.4%). Though other real-world cohorts describe delayed cardiac events related to trastuzumab, our lack of routine long-term cardiac follow-up did not reflect this trend with all four major cardiac events occurring less than 10 months from the end of adjuvant chemotherapy. Even though this is a retrospective real-world analysis with many possible confounders, the optimal sequencing of anthracyclines and taxanes-trastuzumab with respect to cardiac toxicity needs to be further evaluated, especially in the setting of shorter duration trastuzumab.

As with other real-world datasets, this analysis has limitations of its retrospective nature, small sample size, short follow-up duration and missing data points. In patients with locally advanced disease and limited access to trastuzumab, all attempts were made to provide them at least 12 weeks of trastuzumab, which is evident in the selection bias with more advanced disease receiving shorter duration trastuzumab. The relative efficacy and cost effectiveness of 12 weeks of trastuzumab versus no trastuzumab or 1 year of trastuzumab cannot be discerned from this analysis because of base line imbalances with 74% patients having stage III disease in ≤12 weeks cohort. However, compared with the historical controls of patients who have not received any trastuzumab, the use of short duration trastuzumab (≤12 weeks) regimen can be considered as a viable option in resource-constrained settings to improve p-CR and OS. While 1-year trastuzumab remains the standard of care for non-metastatic HER2-positive breast cancer, shorter duration of trastuzumab treatment warrants further evaluation. In this analysis, only one biosimilar (Biocon Biologics Biosimilar) was compared against innovator, which was non-inferior for p-CR outcomes. During the study period, only Biocon Biologics biosimilar was available in the formulary, hence only this biosimilar was used to compare with the innovator. The reason of comparison of innovator versus biosimilar was socioeconomic so that all patients can afford the complete treatment. The biosimilar brand, which was available in the formulary, was the closest to the innovator in terms of safety, efficacy, purity and potency. This biosimilar was approved by the United States Food and Drug Administration (USFDA) and proved equivalence with the innovator in a global large multi-centric randomised trial. In India, over 75% cancer expenses are non-remunerable and out of pocket [31]. Additionally, trastuzumab is not funded by any insurance schemes [32]. The availability of a safe and cost effective biosimilar trastuzumab with proven quality can help more and more patients with advanced disease to access this essential treatment.

## Conclusion

Early introduction of trastuzumab in the neoadjuvant setting in HER2-positive non-metastatic breast cancer improves p-CR. Our results suggest that when the standard 1 year of adjuvant trastuzumab is not feasible, a regimen that incorporates shorter duration of trastuzumab (including biosimilar) delivered concurrently with taxanes and sequentially with anthracyclines is a reasonable alternative. Access to anti HER2 therapy in the treatment of non-metastatic HER2 overexpressed breast cancer in resource-constrained settings has improved significantly with the availability of the biosimilar trastuzumab. This study reports comparable p-CR with the biosimilar trastuzumab versus the innovator. An improvement in DFS and OS in all patients who received trastuzumab was similar to historical data.

While this is the first report of the biosimilar trastuzumab in non-metastatic breast cancer in the neoadjuvant setting, it carries a limitation of being a non-randomised study with retrospective data.

## Conflict of interests

The author(s) declare that they have no conflict of interest.

## Funding

This research received no external funding.

## Figures and Tables

**Table 1.** Patient characteristics.

Characteristic	No trastuzumab *N* = 31	Short duration trastuzumab (≤12 weeks) *N* = 31	Longer duration trastuzumab (>12 weeks) *N* = 73
Trastuzumab duration weeks (mean, range)	nil	10 (2–12)	45 (14–52)
Age (Median)	53 years	50 years	51 years
Female, *n* (%)	31 (96.8%)	31 (100%)	73 (100%)
Male, *n* (%)	1 (3.2%)	-	-
**Menopausal status, *n* (%)**			
Premenopausal	11 (35.5%)	15 (48.4%)	32 (43.8%)
Postmenopausal	20 (64.5%)	16 (51.6%)	41 (56.2%)
**T stage (clinical), *n* (%)**			
Tx	0	1 (3.2%)	1 (1.4%)
T1	2 (6.5%)	1 (3.2%)	5 (6.8%)
T2	13 (41.9%)	5 (16.1%)	30 (41.1%)
T3	5 (16.1%)	4 (12.9%)	10 (13.7%)
T4	11 (35.5%)	18 (58.1%)	27 (37.0%)
**N stage (clinical), *n* (%)**			
N0	10 (32.3%)	6 (19.4%)	15 (20.5%)
N1	15 (48.4%)	13 (41.9%)	30 (41.1%)
N2	6 (19.4%)	10 (32.3%)	22 (30.1%)
N3	-	-	6 (8.2%)
**AJCC stage**, ***n* (%)**			
IA	1 (3.2%)	1 (3.2%)	3 (4.1%)
IB	1 (3.2%)	-	-
IIA	6 (19.4%)	1 (3.2%)	12 (16.4%)
IIB	7 (22.6%)	4 (12.9%)	12 (16.4%)
IIIA	5 (16.1%)	5 (16.1%)	17 (23.3%)
IIIB	11 (35.5%)	18 (58.1%)	23 (31.5%)
IIIC	-	-	6 (8.2%)
**Grade of tumour, *n* (%)**			
Grade 1	-	2 (6.5%)	3 (4.1%)
Grade 2	13 (41.9%)	18 (58.1%)	33 (45.2%)
Grade 3	18 (58.1%)	11 (35.5%)	37 (50.7%)

**Table 2.** Treatment details.

Treatment characteristics	*n*	(%)
**Histopathology of tumour**		
Ductal carcinoma	135	100
**Anti-HER2 therapy**		
Received any trastuzumab (innovator + biosimilar)	104	77
Biosimilar trastuzumab	70	52
Did not receive trastuzumab	31	23
**Chemotherapy regimens**		
Anthracycline + cyclophosphamide → Paclitaxel + trastuzumab	87	64.4
Anthracycline + cyclophosphamide → Paclitaxel	28	20.7
Paclitaxel + trastuzumab	8	5.9
Taxane + carboplatin + trastuzumab	6	4.4
Anthracycline + cyclophosphamide	3	2.2
Anthracycline + cyclophosphamide+ trastuzumab	2	1.5
Trastuzumab + letrozole	1	0.7
**Chemotherapy dosing frequency (*N* = 40)**		
Dose-dense (every 2 weeks)	60	44.4
Every 3 weeks	75	55.6
**Growth factor support**		
Peglylated GCSF given	113	83.7
No GCSF support	22	16.3

**Table 3.** Pathological assessment post neoadjuvant treatment.

Pathological response	*n* (*N*)	%
**p-CR (ypT0/is N0)**		
p-CR with neoadjuvant chemotherapy and trastuzumab	26 (69)	37.6
p-CR with neoadjuvant chemotherapy and innovator trastuzumab	6 (21)	28.5
p-CR with neoadjuvant chemotherapy and biosimilar trastuzumab	20 (48)	41.7
p-CR with neoadjuvant chemotherapy without trastuzumab	4 (18)	22.2
p-CR overall	30 (87)	34.5
Breast p-CR (ypT0/is N0/+)	42	40.3
Nodal p-CR (ypN0)	60	57.7
*N*, Total number of patients evaluated for p-CR; *n*, Number of patients achieving p-CR		
Miller Payne	86	
Miller Payne grade 1	3	3.5
Miller Payne grade 2	8	9.3
Miller Payne grade 3	7	8.1
Miller Payne grade 4	26	30.2
Miller Payne grade 5	42	48.8
